# A high-resolution daily climate time series for Germany (1981-2100)

**DOI:** 10.1016/j.dib.2026.113218

**Published:** 2026-08-30

**Authors:** Kilian Hochholzer, Christina Dollinger, Marc Grünig, Rupert Seidl, Werner Rammer

**Affiliations:** a Technical University of Munich, TUM School of Life Sciences, Ecosystem Dynamics and Forest Management Group, Hans-Carl-von-Carlowitz-Platz 2, 85354 Freising, Germany; b Berchtesgaden National Park, Doktorberg 6, 83471 Berchtesgaden, Germany; c Swiss Tropical and Public Health Institute (Swiss TPH), Kreuzstrasse 2, 4123 Allschwil, Switzerland; d University of Basel, Petersplatz 1, 4001 Basel, Switzerland; e Chair of Modelling of Social-Ecological Systems, University of Freiburg, Tennenbacher Str. 4, 79106 Freiburg im Breisgau, Germany

**Keywords:** Climate time series, Downscaling, Spatial clustering, EURO-CORDEX

## Abstract

The presented dataset is a high-resolution (1 × 1 km) daily climate time series for Germany covering the period 1981–2100 across a historic and nine CMIP5 climate change scenarios. The dataset is based on EURO—CORDEX projections from three Earth system models (ICHEC-EC-EARTH, MPI-M-ESM-LR, and NCC—NorESM1-M) under three representative concentration pathways (RCP 2.6, 4.5, and 8.5) and was bias-corrected against observational data from the German Weather Service (DWD). To reduce data volume in climate impact studies, the 355,944 1 × 1 km grid cells were aggregated into 10,000 spatial clusters based on multi-annual averages of five key variables for plant growth, specifically, temperature (minimum and maximum), precipitation, global radiation and vapor pressure deficit. The resulting dataset provides for each cluster nine daily climate time series of said variables until the end of the 21st century. The dataset is stored in SQLite databases and accompanied by a map relating representative climate clusters to the 1 km grid cells. To our knowledge, this is the first Germany-wide dataset that provides ready to use daily climate projections until 2100 at 1 km resolution with a climate-cluster representation that reduces computational demand, while keeping climatic variability.

Specifications Table

**Table:** 

Subject	Earth & Environmental Sciences
Specific subject area	Climate science / environmental science, with contributions from data science
Type of data	SQLite-Databases, raster, R and Python Scripts Data format: Processed (SQLite-databases), raster data (weather service raster)
Data collection	Daily climate data were derived from German Weather Service (DWD) 1 km gridded observations (1981–2010) and EURO—CORDEX regional climate model outputs. Variables were harmonized, aligned to EPSG:31,467, and vapor pressure deficit was calculated from temperature and humidity. Data volume was reduced through clustering (10k clusters) using MiniBatchKMeans (scikit-learn). Bias correction used additive (temperature) and multiplicative (others) adjustments against DWD data. Data stored as SQLite time-series databases.
Data source location	The data were collected over Germany using gridded observations from the German Weather Service (DWD) Climate Data Center at 1 × 1 km resolution (EPSG:31,467). Additional data originate from EURO—CORDEX regional climate projections based on CMIP5 models. The processed dataset is stored as SQLite databases containing cluster based daily time series for the entire German territory (1981–2100).
Data accessibility	Repository name: Zenodo Data identification number: 10.5281/zenodo.20201156 Direct URL to data: https://zenodo.org/records/20201156 Instructions for accessing these data: Download the .zip files from the repository, these contain .sqlite files with the data inside.
Related research article	none

## 1 Value of the Data

• Climate data at high resolution are useful for a variety of applications in different fields like forestry, agriculture, ecology, infrastructure, and urban planning. A spatial resolution exceeding that of typical regional climate models (i.e., 12.5 km) is necessary to resolve environmental gradients in complex terrain, for example mountainous and hilly landscapes.
• Daily temporal resolution is needed to capture biological responses to extreme events, which have important implications e.g., for phenology [1,2], drought [3,4], disturbances [5], or species distribution [6].
• The primary purpose of this dataset is to provide a daily climate ensemble for Germany that is ready-to-use for climate impact science. While applicable across various disciplines, the data are natively formatted for direct integration into the process-based forest landscape model iLand [9].
• The dataset delivers continuous daily records for five meteorological variables (see Table 1) from 1 January 1981 to 31 December 2100. A reference raster is provided to link geographic locations to the corresponding climate information.

**Table 1:** Climate parameters and their descriptions.

Parameter	Unit	Description
Year		The absolute year (e.g., 2026)
Month		Month of the year, where January = 1 and December = 12
Day		Day of the month, from 1 to 31. February consists of 28 days in non-leap years and 29 in leap years
Maximum temperature	°C	Maximum temperature of the day
Minimum temperature	°C	Minimum temperature of the day
Precipitation	mm	Precipitation sum of the day
Global radiation	MJ m^−2^day^−1^	Daily sum of global radiation per square meter
Vapor pressure deficit	kPa	Average vapor pressure deficit of the day
• The dataset includes information on both historical trajectories and future projections from three global circulation models driven by CMIP5 climate scenarios: ICHEC-EC-EARTH, MPI-M-ESM-LR, and NCC—NorESM1-M, all regionalized with the regional climate model SMHI-RCA4. For each model, data are available for three Representative Concentration Pathways (RCP 2.6, 4.5, and 8.5), enabling the consideration of both climate model uncertainty and scenario uncertainty in analyses. To ensure realism and consistency across historical and future conditions, the dataset was bias corrected against historically observed reference data from the German Weather Service.

## 2 Background

Climate change is fundamentally affecting ecosystems worldwide, increasingly challenging their ability to provide ecosystem services to society. Future climate projections are needed for a broad range of applications ranging from crop and irrigation planning [7,8], ecosystem modelling [9], and species distribution modelling [6,10,11] to infrastructure planning [12,13].

The five climate variables at the center of this dataset are a subset of essential climate variables that due to having information in addition to temperature and precipitation allow for greater insight into biological and Earth system processes [20].

For Europe, the EURO—CORDEX initiative provides high-quality regional climate projections from multiple climate models and across various Representative Concentration Pathway (RCP) scenarios. However, the modelled EURO—CORDEX datasets are limited in their spatial resolution to approximately 12 km, requiring downscaling and local bias correction for studies that demand finer spatial details (see Fig. 3). The downside of higher resolution climate data, however, is the strongly increasing data volume, which can create challenges in data handling and modeling (e.g., memory issues).

Our goal was to provide high-resolution climate data for Germany, and to use clustering to significantly reduce the effective data volume while retaining most of the information content of the downscaled data set. Increasing the resolution to 1 km improves the representation of local climatic gradients and regional variability, thereby improving the utility for various applications. Clustering the downscaled climate grids retains most of the high-resolution information, while resulting in a more manageable dataset, improving the experience for data users.

## 3 Data Description

The dataset includes a 1 × 1 km reference grid over Germany (EPSG 31,467). This grid contains a *ClusterID*, i.e. a numeric value linking one of the 10,000 unique climate clusters to a given grid cell. The climate data is organized as one SQLite database for historic and each of the three climate scenarios (RCP 2.6, 4.5, 8.5) per global circulation model (ICHEC-EC-EARTH, MPI-M-ESM-LR, and NCC—NorESM1-M), totaling 12 SQLite databases. Each database holds a table with a time series of climate variables for each *ClusterID*, i.e., a total of 10,000 tables per database.

Each table includes columns for year, month, day, minimum and maximum temperature, precipitation, radiation, and vapor pressure deficit (see Table 1). The dataset contains for each of the three climate models a database with historical data (1980–2005), and three databases for Representative Concentration Pathways (2.6, 4.5, 8.5) spanning 2005 to 2100. The clustering approach helps to decrease the dataset size by a factor of about 35, resulting in file sizes of 5.4 and 20.4 GB, for historic and RCP scenarios, respectively. The naming convention of the SQLite databases reflects the global circulation model and RCP scenario used, which is as follows: “*climate_db_<climate model name>_<RCP scenario>.sqlite*” (for example “*climate_db_ICHEC-EC-EARTH_rcp2_6.sqlite*”). SQLite databases were used instead of NetCDF files, due to their ease of use with different programming languages (R, Python) used by the climate impact community. A practical example of working with the structure of the data set is provided in the “Example Usage” section below.

## 4 Experimental Design, Materials and Methods

**Fig. 1:**
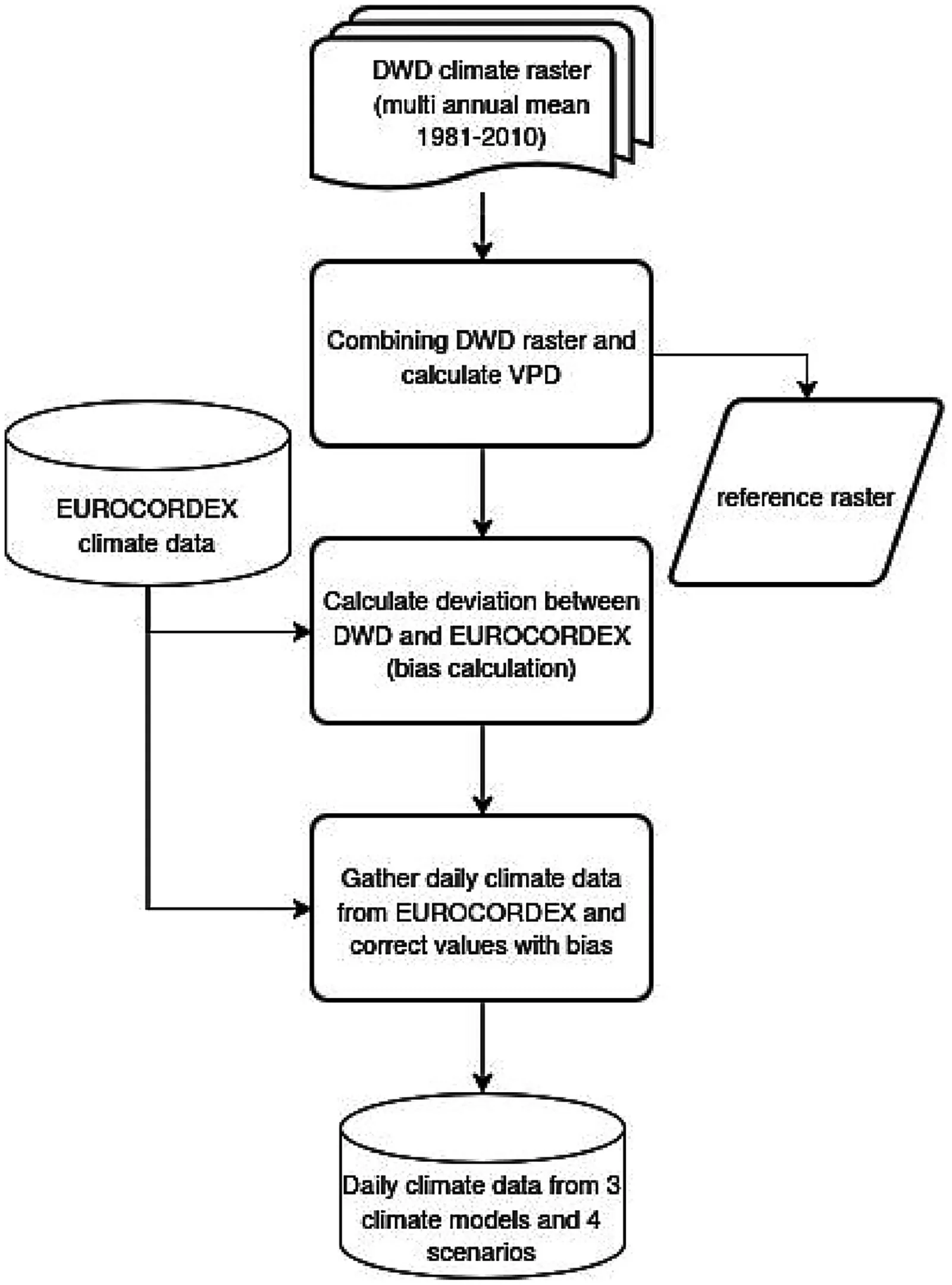
Flowchart of workflow, showing inputs and outputs.

This dataset combines observational climate data from the German Weather Service (DWD), as provided by the DWD Climate Data Center (CDC) [[14], [15], [16], [17], [18], [19]] (downloaded at: https://opendata.dwd.de/climate_environment/CDC/grids_germany/multi_annual/), with regional climate projections from EURO—CORDEX (downloaded at https://cds.climate.copernicus.eu/datasets/projections-cordex-domains-single-levels?tab=overview) to generate a high-resolution (1 × 1 km), bias-corrected daily climate dataset for Germany. The workflow involves (i) acquiring and preprocessing DWD climate variables, (ii) performing a clustering of Germany at 1 × 1 km resolution based on DWD climate variable, and (iii) perform bias-correction of the time series from EURO—CORDEX to the empirical reference from DWD for each climate cluster (see Fig. 1 for an overview of the workflow).

### 4.1 Data acquisition and pre-processing

Gridded multi annual datasets derived from measurements at weather stations and subsequent interpolation, between 1981 and 2010, were accessed through the DWD Climate Data Center [[14], [15], [16], [17], [18], [19]]. The data sourced from DWD included daily mean, minimum and maximum temperatures, relative humidity, precipitation, and global radiation. All datasets were available as raster layers at 1 × 1 km spatial resolution. These variables served both as input for determining homogeneous climate clusters and as observational reference for bias-correction. All layers were harmonized to the Gauss-Krueger coordinate reference system (EPSG:31467) and spatially aligned to the German 1 × 1 km grid.

Next, the daily vapor pressure deficit (VPD) was calculated from near-surface air temperature (daily mean temperature from DWD) and near-surface relative humidity sourced from DWD. VPD was derived following the psychrometric formulation based on the difference between saturation vapor pressure at air temperature and at dew point temperature. Saturation vapor pressure e_S_ (kPa) at temperature T ( °C) was calculated using Eq. 1:

(1) $$e_{S}\left(T\right)=0.611*exp\left(17.67*T/T+243.5\right)$$

Daily VPD was then computed via Eq. (2):

(2) $$VPD=e_{S}\left(T\right)-e_{S}\left(T_{d}\right)$$

where T is the daily mean air temperature (°C), T_d_ is the daily mean dew point temperature (°C), derived from relative humidity, and 0.611 is the saturation vapor pressure at 0 °C in kPa. All VPD values were constrained to non-negative values, and calculations were implemented using the *weathermetrics* R package. All VPD values have kPa as unit. Computed VPD values were then used as one of the input variable for clustering.

### 4.2 Clustering

Storing daily climate information for every 1 × 1 km grid cell across Germany would require a high level of storage resources – with eight variables an estimated 7 TB. However, many areas, especially those with low climatic heterogeneity such as the North German Plain, have low spatial variation in daily climate. To reduce data volume while preserving spatial climate heterogeneity, a clustering approach was applied to group areas with similar long-term climate characteristics.

Clustering was performed on the 1 × 1 km DWD data with a k-means clustering, specifically using the *MiniBatchKMeans* algorithm from the *scikit-learn* library in Python. Input variables included mean multi-annual (1981–2010) maximum and minimum temperature, precipitation, global radiation, and VPD. Variables were standardized prior to clustering, using the function *sklearn.preprocessing.StandardScaler* with default settings.

**Fig. 2:**
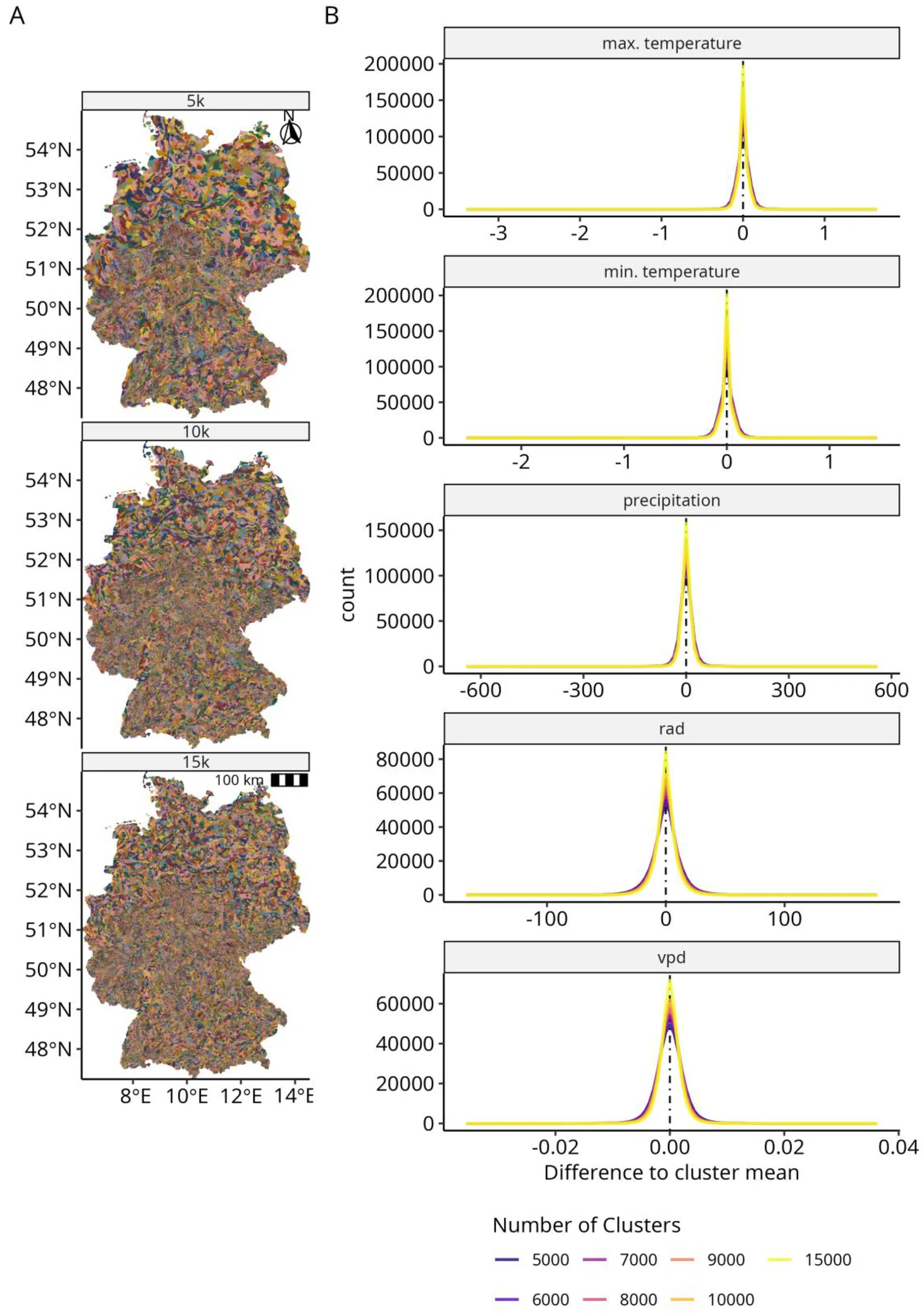
Spatial distribution of climate clusters and density distribution of residual multi-annual mean values. (A) Climate clusters for k = 5000, 10,000, and 15,000, with colors indicating cluster IDs (12-color modulo scheme). (B) Residual value densities for cluster sizes between 5000 and 15,000, showing largely consistent distribution across clustering resolutions.

Evaluation of k (i.e., the number of clusters) was done for values ranging from 5000 to 15,000 (i.e., 1.4% to 4.2% of the full 1 × 1 km dataset) to identify an optimal balance between spatial detail, within-cluster homogeneity, and storage efficiency. Criteria for evaluation included preservation of climatic gradients across Germany, cluster compactness, and computational cost. Based on these metrics and visual inspection, 10,000 clusters were selected as a good compromise (see Table 2, Fig. 2).

**Table 2:** Mean residuals (± SD), storage requirement and mean cluster area (± SD) for all climate cluster sizes. Residuals are the average deviation of 1 km cells from cluster centroids. Storage requirements refer to the SQLite databases containing the daily climate data for all scenarios across Germany.

Number of clusters	Residual multi-annual maximum temperature [ °C]	Residual multi-annual minimum temperature [ °C]	Residual multi-annual precipitation [mm]	Residual multi-annual global radiation [MJm-2day-1]	Residual multi-annual vapour pressure deficit [kPa]	Storage requirement [GB]	Mean cluster area [km²]
5k	−0.000 ± 0.077	0.000 ± 0.070	0.029 ± 18.524	−0.010 ± 12.472	−0.000 ± 0.002	95.5	71.189 ± 51.007
6k	−0.000 ± 0.074	−0.000 ± 0.066	0.013 ± 17.535	−0.002 ± 11.812	−0.000 ± 0.002	115	59.324 ± 42.796
7k	−0.000 ± 0.069	−0.000 ± 0.063	0.002 ± 16.818	−0.001 ± 11.264	−0.000 ± 0.002	134	50.849 ± 36.544
8k	−0.000 ± 0.067	−0.000 ± 0.060	0.006 ± 16.296	−0.001 ± 10.847	−0.000 ± 0.002	153	44.493 ± 31.559
9k	−0.000 ± 0.064	0.000 ± 0.058	0.010 ± 15.773	−0.004 ± 10.484	−0.000 ± 0.002	172	39.549 ± 26.959
10k	−0.000 ± 0.062	0.000 ± 0.056	0.016 ± 15.217	0.003 ± 10.093	−0.000 ± 0.002	191	35.594 ± 23.801
15k	−0.000 ± 0.054	0.000 ± 0.049	0.016 ± 13.546	−0.005 ± 8.915	0.000 ± 0.002	287	23.730 ± 15.060

**Fig. 3:**
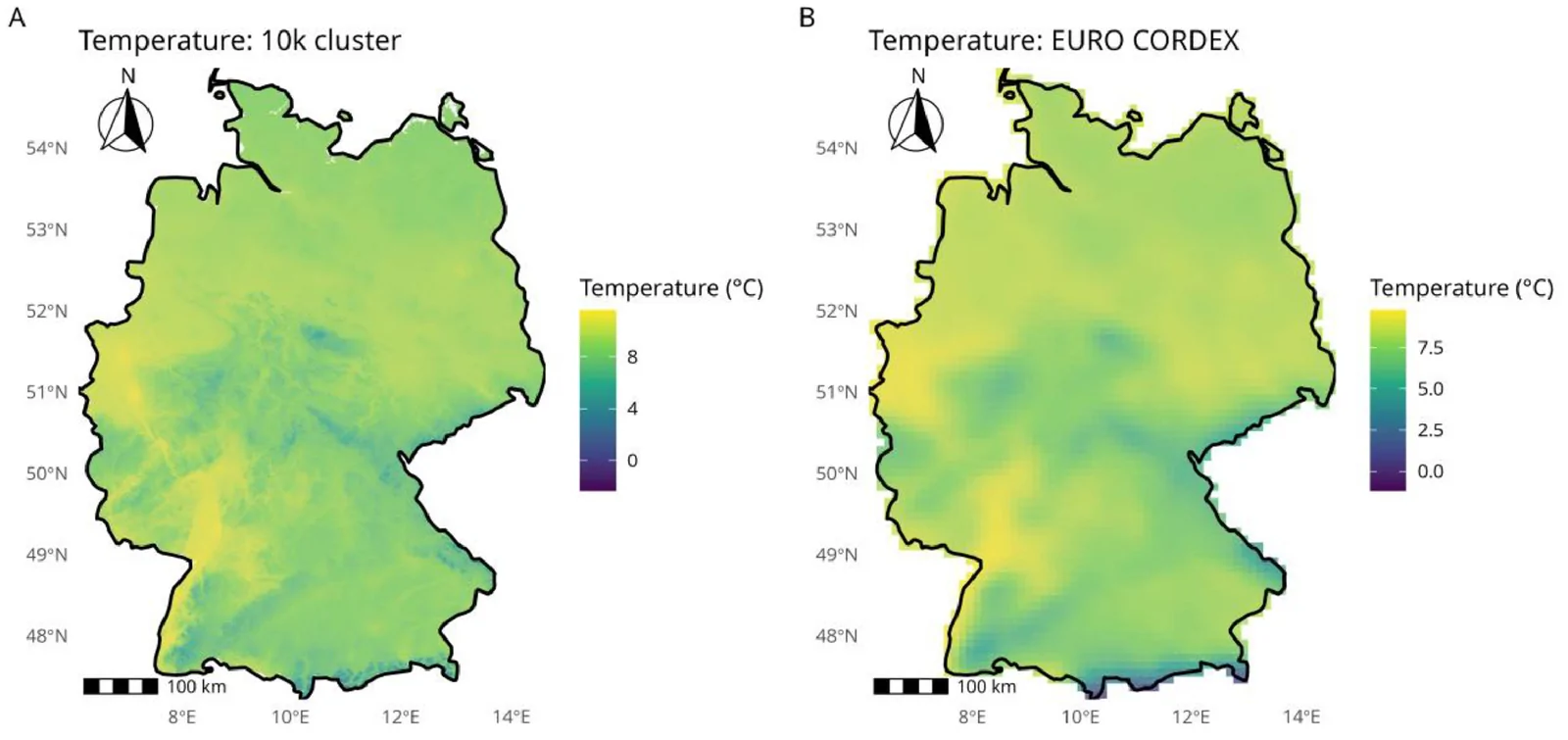
Difference in granularity between 1 km and 12 km resolution, illustrated for temperature. (A) depicts historic average temperature from 10,000 clusters. (B) shows mean historic average temperature from EURO—CORDEX at ∼12 km resolution.

### 4.3 Cluster evaluation

Cluster evaluation focused on assessing internal homogeneity and spatial coherence. Within-cluster variability (compression error) was evaluated for each of the 10,000 final clusters by calculating the mean, standard deviation, relative variability (coefficient of variation), and difference in value between point in a cluster and the cluster mean of climate variables and cluster area.

Climate variables showed overall low within-cluster variability, indicating a high degree of within-cluster homogeneity. Relative variability ranged from 0 to 0.1 for VPD, 0 to 0.12 for precipitation, 0 to 0.02 for global radiation and 0 to 0.98 for maximum temperature. This identifies within cluster variability as very low for global radiation, low for VPD and precipitation, and as highest for maximum temperature.

**Fig. 4:**
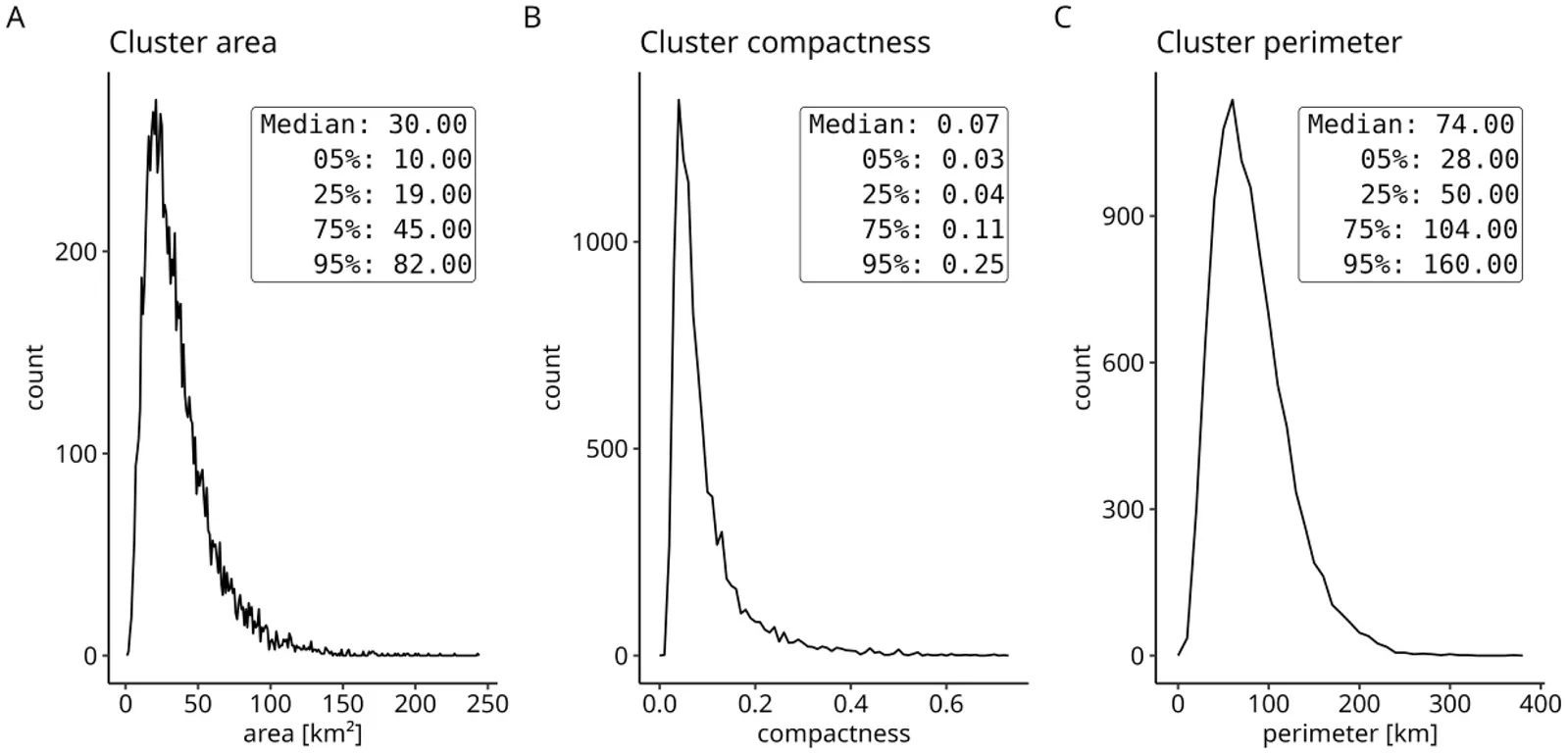
Histograms showing the distribution of (A) cluster area, (B) cluster compactness and (C) cluster perimeter. Compactness values indicate shape of cluster where 0.785 would be a perfect square, 1 a circle and close to 0 highly elongated or irregularly shaped. Most clusters are below 50 km² with irregular shapes. Perimeter = distance around the outside of the cluster areas.

Cluster spatial characteristics were evaluated using area, compactness, and cluster perimeter derived from the 1 × 1 km grid. Cluster size averaged 35.59 ± 23.8 km², with sizes ranging from 2 km² to 243 km², suggesting a resolution suitable for spatial regionalization. Cluster compactness indicated irregular or elongated shapes, visible in mean compactness of 0.09 (minimum 0.01, maximum 0.72; Fig. 4B), reflecting topographic features (e.g., such as valleys) and their effect on spatial heterogeneity in climate patterns. The average cluster perimeter was 80.91 km, with values ranging from 6 km to 366 km.

### 4.4 Bias correction of time-series

For each cluster, we selected as representative cell the grid cell closest to the cluster mean using minimum Euclidean distance, based on variables in their original units. This representative cell was then linked to the EURO—CORDEX grid cell at the same location. The deviation in mean values for 1981–2010 between DWD and EURO—CORDEX were subsequently used to derive variable-specific bias correction factors.

Correction factors were computed from the multi annual means separately for each of the five climate variables and for every climate model. Additive correction T_bias_=T_DWD_-T_CORDEX_ was used for temperature variables. Precipitation, global radiation and VPD were adjusted using multiplicative corrections P_bias_=P_DWD_/P_CORDEX_. Derived correction factors were applied to the full EURO—CORDEX time series (1981–2100) for each model and RCP scenario combination, producing bias-adjusted daily climate values for each representative cell and thus cluster. For example, for temperature T_corrected_=T_cluster_+T_bias_ and precipitation P_corrected_=P_cluster_*P_bias_.

All corrected daily climate data were then stored in 12 SQLite databases. Each database corresponds to one climate model-scenario combination, plus a historical database for each model. Quality checks included verification of variable ranges, inspection of cluster homogeneity, and evaluation of bias-corrected EURO—CORDEX data against DWD observations.

### 4.5 Example usage

Querying the database for a specific location is done in three steps: (1) locate coordinates or area in the reference raster and identify corresponding climate clusters, (2) gather climate data from the respective climate clusters from the relevant SQLite database representing the desired climate model and scenario, and (3) save, analyze or apply the gathered climate data. A full example usage R Markdown file can be found in the repository (4_use/example_use.Rmd).

## Limitations

We used gridded multi annual averages based on historical observations as ground truth for the downscaling. These grids were created by DWD through interpolation between weather stations. The data therefore includes uncertainties both from the weather stations measurements itself and the spatial interpolation. For the downscaling we assumed stationarity of the statistical relationship between GCMs/RCMs and local conditions, which might not hold for future climate conditions. Similarly, statistical downscaling can over- or underestimate local effects under strong future forcing. While this applies to virtually all statistical downscaling methods used for climate change impact studies, the clustering approach adds additional uncertainty. The clustering significantly reduces the amount of data, and thus the required memory and compute, when using the climate data. However, this data “compression” step inevitably reduces local accuracy (see Fig. 2).

**Fig. 5:**
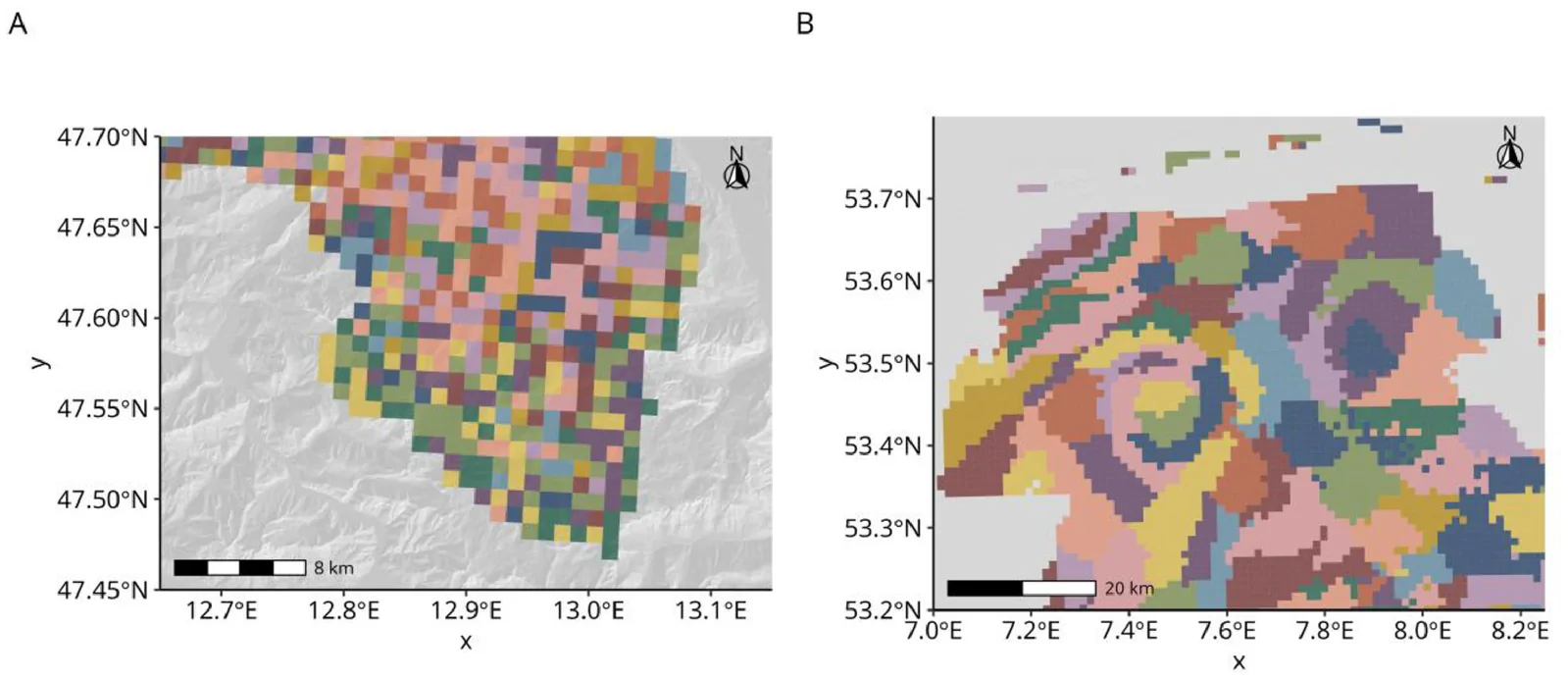
Climate clusters for the regions of (A) Berchtesgaden, in the south-eastern German Alps and (B) east Frisia, at the north-western German coast to the North Sea. Colors representing cluster IDs after modulo division (i.e., 12 distinct colors) The mountainous region of Berchtesgaden (A) shows very small climate cluster regions, while in the topographically simpler case of east Frisia (B) the cluster regions are considerably bigger. Panels A and B have different extents but same orientation and grain.

While a 1 × 1 km resolution is sufficient for many uses, it might still be too coarse for some applications. Particularly for topographic diverse landscapes such as mountains, local climatic gradients might not be sufficiently captured, requiring higher-resolution data or additional downscaling (see Fig. 5).

The dataset contains daily climate data from three global circulation models from the CMIP5 series released more then 10 years ago. While newer GCM model runs exist (e.g., from CMIP6), regionalized climate projections (RCM) used here for the downscaling approach are not yet available. Moreover, future climate projections inherently rely on modelled data, and do not represent real observed values.

## Ethics Statement

The authors state that they have read and follow the ethical requirements for publication in Data in Brief and confirm that the current work does not involve human subjects, animal experiments or any data collected from social media platforms.

## CRediT Author Statement

**KH:** Data curation, formal analysis, investigation, methodology, software, validation, visualization, writing – original draft. **CD:** Methodology, software, writing – review and editing. **MG:** Data curation, methodology, software, writing – review and editing. **RS:** Conceptualization, resources, writing – review and editing, funding acquisition. **WR:** Conceptualization, project administration, resources, supervision, validation, writing – review and editing, funding acquisition

## Declaration of Generative AI and AI-assisted Technologies in the Manuscript Preparation Process

During the preparation of this work the main author used Microsoft Copilot in order to help improve the flow and readability of the initial manuscript. After using this tool, the authors reviewed and edited the content as needed and take full responsibility for the content of the published article.

## Data Availability

ZenodoData for: A high-resolution daily climate time series for Germany (1981–2100) (Original data)
